# Longitudinal pathways between parent depression and child mental health in families of autistic children

**DOI:** 10.1017/S0954579424001378

**Published:** 2024-09-23

**Authors:** Brianna Piro-Gambetti, Jessica Greenlee, Daniel Bolt, Kristin Litzelman, Sigan L. Hartley

**Affiliations:** 1 School of Human Ecology, University of Wisconsin-Madison, Madison, WI, USA; 2 Waisman Center, University of Wisconsin-Madison, Madison, WI, USA; 3 Department of Psychology, Lafayette College, Easton, PA, USA; 4 School of Educational Psychology, University of Wisconsin-Madison, Madison, WI, USA

**Keywords:** Autism, behavior problem, depression, mental health, parent–child

## Abstract

Autistic children and their parents are at risk for mental health problems, but the processes driving these connections are unknown. Leveraging three data cycles (spaced *M* = 11.76 months, *SD* = 2.77) on 162 families with autistic children (aged 6–13 years), the associations between parent–child relationship quality (warmth and criticism), child mental health problems, and parent depression symptoms were examined. A complete longitudinal mediation model was conducted using structural equation modeling. Father depression mediated the link between child mental health problems and father critical comments (*β* = −0.017, *p* = 0.018; CI [−.023 – −.015]). Father report of child mental health problems mediated the association between father depression and father critical comments (*β* = 0.016, *p* = 0.040; CI [0.003–0.023]) as well as the association between father positive remarks and father depression (*β* = −0.009, *p* = 0.032; CI [−0.010 – −0.009]). Additionally, father positive remarks mediated the connection between father depression and child mental health problems (*β* = 0.022, *p* = 0.006; CI [0.019–0.034]). No mediation effects were present for mothers. Findings highlight that the mental health of parents and autistic children are intertwined. Interventions that improve the parent–child relationship may reduce the reciprocal toll of parent and child mental health problems.

Autism spectrum disorder is a neurodevelopmental condition that occurs in approximately 1 in 36 children in the United States, with boys four times more likely to receive this diagnosis than girls (Centers for Disease Control and Prevention, 2023, 2022). Autism involves restricted and repetitive behaviors and differences in social communication (American Psychological Association, 2020). Autistic children face a high risk for mental health problems compared to neurotypical children (Boss et al., 2018; Hudson et al., 2019; Vasa & Mazurek, 2015). Indeed, it is estimated that 70%–95% of autistic children experience mental health conditions such as depression, anxiety, or attention deficit hyperactivity disorder (Joshi et al., 2010; Lever & Geurts, 2016; Mosner et al., 2020). Parents of autistic children also experience a high risk for mental health problems, with approximately 31%–49% of parents of autistic children reporting clinically significant depression (Al-Farsi et al., 2016; Schnabel et al., 2020). Parent depression and child mental health problems are linked in bidirectional ways within families of both neurotypical and autistic children (e.g., Benson, 2018; Goodman et al., 2010; Letourneau et al., 2010; Piro-Gambetti et al., 2022). For example, Goodman et al. (2010) found that children of depressed mothers have higher rates of internalizing mental health problems than children of nondepressed mothers by middle school. Child mental health problems (in both neurotypical and autistic samples) have also been associated with an increase in parent depression symptoms over time (Benson, 2018; Piro-Gambetti et al., 2022; Stone et al., 2016). Tracking a sample of 110 mothers of autistic children over 12 years, Benson (2018) reported that increased child-related stressors (i.e., higher severity of child behavior problems) led to increased maternal depression. The underlying mechanisms that drive the association between parent depression and child mental health, however, are less clear.

Within a family systems framework, the transactional exchanges that occur among family members are posited to shape child and parent development (Cox & Paley, 1997). It is thus through everyday parent–child interactions that the mental health of parents is thought to influence the mental health of the child and vice versa (Cox & Paley, 1997; Cox & Paley, 1997; Cridland et al., 2014; Kerig, 2016). In the context of child autism, both parents and the autistic child may share biological predispositions for mental health problems. Indeed, mothers of autistic children are at increased risk for depression with the onset of symptoms prior to having the autistic child (Hagberg et al., 2018; Vasa et al., 2012; Wiggins et al., 2019). Moreover, maternal depression before becoming a parent is associated with having an autistic child with greater emotion dysregulation problems (Wiggins et al., 2019). These biological predispositions, however, are thought to interact with the quality of the parent–child relationship to alter parent–child transactional exchanges to shape the mental health of parents and the child (e.g., Cummings et al., 2000), with effects flowing in both directions.

There is marked evidence that parent mental health alters the quality of the parent–child relationship. Indeed, frequent exposure to parent depression symptoms and recurrent and/or severe parent depressive episodes predicts increases in child mental health problems, especially internalizing problems (e.g., mood problems), for neurotypical children (Kamis, 2021; Schnabel et al., 2020; Tirumalaraju et al., 2020) and autistic children (Cohen & Tsiouris, 2006; Wiggins et al., 2019). Depressed parents often struggle to engage in sensitive and responsive parenting (Letourneau et al., 2010; Taraban & Shaw, 2018), instead resorting to harsher, more disengaged, and critical parenting (Wilson & Durbin, 2010). Depressed mothers report less sensitive, warm, responsive, and consistent parenting strategies relative to nondepressed mothers (Bayer et al., 2006; Goodman et al., 2010; Rueger et al., 2011), and similar results have been found with depressed fathers (Wilson & Durbin, 2010). Consistent exposure to these types of maladaptive parent–child interactions predicts later child mental health problems (Aktar & Bögels, 2017; Goodman & Gotlib, 1999; Goodman, 2020).

In a transactional manner, child mental health problems also shape the quality of the parent–child relationship. In general population research, child externalizing mental health problems are related to a lower quality parent–child relationship through elevated parenting stress (Mackler et al., 2015). Research on families with autistic children has revealed similar findings. McRae et al. (2018) assessed parent depressive and anxiety problems, parenting behaviors, and child mental health problems in 67 parents of autistic children aged 6–12 years. They found that parent depressive and anxiety problems predicted more harsh and disengaged parenting and greater child internalizing mental health problems. In turn, more harsh and disengaged parenting predicted greater child externalizing problems, while warmer and more supportive parenting predicted fewer child internalizing problems (McRae et al., 2018). Similarly, Hickey et al. (2020), using a sample of 150 parents with autistic children aged 5–12 years, found that mothers who self-reported fewer depression symptoms and less parenting stress exhibited more warmth and less criticism toward their child in a five-minute speech sample (FMSS). Fathers who self-reported less parenting stress exhibited less criticism toward the child in their FMSS. Together, these findings highlight the potential mediating role of emotional quality of the parent–child relationship in driving the connection between parent and child mental health problems.

The primary goals of the current study were to investigate the longitudinal associations between parent depression symptoms, parent–child relationship quality, and child mental health problems. The primary association of interest was whether the parent–child relationship quality (assessed by conveyed warmth and criticism toward the autistic child during a FMSS) mediates an association between parent depression symptoms and child mental health problems in families with autistic children. However, we also modeled and assessed other direct and mediation pathways (i.e., parent depression mediator of the association between child mental health and parent–child relationship quality) that may be occurring. Analyses drew on a longitudinal study of families of autistic children (age 6–13 years) involving three time points of data collection, each spaced approximately 12 months apart (*M* = 11.76 months, *SD* = 2.77). Parents completed questionnaires about their own and their child’s mental health and a FMSS was used to assess warmth and criticism in the parent–child relationship.

## Study aims and hypotheses

The study aims were to: (1) determine the association between parent–child relationship quality and parent depression symptoms and child mental health problems; and (2) evaluate whether parent–child relationship quality serves as a mediator of the association between parent depression symptoms and child mental health problems. Based on a family systems framework and previous research, we hypothesized: (1) higher parent depression symptoms would predict lower warmth and higher criticism in the parent–child relationship 12 months later; (2) higher child mental health problems would predict lower warmth and higher criticism in the parent–child relationship 12 months later; and (3) warmth and criticism in the parent–child relationship would mediate the association between parent depression symptoms and child mental health problems across time. Modifiable aspects of the family environment – such as parent–child relationship quality – that underly the connection between parent and child mental health offer a meaningful intervention target.

## Methods

The present study drew data from the second to the fourth time point of data collection of the Family Outcomes in Autism Spectrum Disorder study, which received IRB approval through the University of Wisconsin-Madison. For the present analyses, we have labeled these time points T1, T2, and T3. At T1, 162 mothers and 156 fathers within the same family (hereby referred to as a parent-couple) with at least one autistic child participated in the study. Using a convenience sampling approach, we recruited participants by distributing fliers throughout community settings, schools, and ASD clinics. Study information was also provided through research registries. The autistic child had to be between 6 and 13 years of age for the purpose of the present analyses and had to have an educational or medical diagnosis of autism spectrum disorder based on a diagnostic evaluation that included the autism diagnostic observation schedule (ADOS-2^nd^ edition; Lord et al., 2012). Seventeen families (10%) had one additional autistic child, and 3 (2%) families had two additional autistic children. If a family had more than one autistic child, the oldest child within the 6–13 year range was considered the target child (i.e., focus of the study). Parents had to be at least 21 years of age or older, live in a cohabiting couple relationship for at least 3 years, and both parents had to agree to participate in the study. To assess the child’s current autism symptoms, parents completed the Social Responsiveness Scale- 2^nd^ Edition (SRS-2; Constantino & Gruber, 2012). All but five children had an SRS-2 total t-score ≥ 60. However, after review of medical/educational records and ADOS scores, these children were deemed to meet criteria for autism spectrum disorder and were included in analyses. Table 1 provides additional family sociodemographics.

### Procedure

At each time point, parent-couples completed a 2.5-hour lab or home visit in which they answered sociodemographic questions and then separately completed questionnaires assessing their own parent depression symptoms and their child’s mental health problems. Additionally, within families, mothers and fathers separately completed a FMSS where they were asked to speak about their child and the parent–child relationship for five minutes. Each parent was compensated $50 for this portion of the study. All parents provided informed consent.

### Measures

#### Family sociodemographics

Parent-couples jointly reported on family sociodemographics, including both parent information (e.g., gender, age, household income), and child information (e.g., biological sex, and intellectual disability (ID) status). Parent gender was coded as mothers = 1, fathers = 2. Parent age was coded in years. Household income was coded on a scale of 1–14 (1 = $1–$9,999, 14 = $160,000+). Parent-reported child ID status was coded as 0 = no ID, 1 = ID and determined by either the presence of a medical diagnosis of ID or if the child met the criteria for ID through IQ testing. Parents also reported on family size and the number of additional autistic children in the family.

#### Parent depression symptoms

Mothers and fathers separately completed the Center for Epidemiological Studies-Depression Scale (CES-D; Radloff, 1977). This 20-item questionnaire involved rating each item on a 4-point scale (e.g., 0 = rarely or none of the time to 3 = most or all of the time). A CES-D total score ≥ 16 suggests clinically significant depression symptoms (Radloff, 1977). Example items include “I was bothered by things that usually don’t bother me” and “I felt that people disliked me.” In the current sample, the CES-D indicated high internal consistency across time in mothers (Cronbach’s *α* = .92–.93) and fathers (Cronbach’s *α* = .91–.93). Table 2 provides the means and standard deviations for mother- and father-reported CES-D total scores across time as well as *t*-values for paired samples *t*-tests.

#### Child mental health problems

Child mental health problems were assessed via the preschool form (ages 1.5–5 years) and the school age form (ages 6–18 years) of the Child Behavior Checklist (CBCL; Achenbach & Rescorla, 2000, 2001). The CBCL is comprised of 113 items in which parents separately rate each item on a 3-point scale (0 = not true (as far as you know) to 2 = very true or often true). The item scores are then summed into a total problems score. The total problems score encompasses eight different syndrome scales: (1) anxious/depressed; (2) withdrawn depressed; (3) somatic complaints; (4) social problems; (5) thought problems; (6) attention problems; (7) rule-breaking behavior; and (8) aggressive behavior. Previous research has indicated that the CBCL has strong reliability in the ASD population (Pandolfi et al., 2014). In the present study, the CBCL had high internal consistency for both mothers (Cronbach’s *α* = .94) and fathers (Cronbach’s *α* = .94–.95). Means and standard deviations for the CBCL total *t*-scores and *t*-scores comparing mother- and father reports are in Table 2.

#### Parent–child relationship quality

At T1-T3, parents separately completed a FMSS (Magaña et al., 1986) in which they spoke about their autistic child for 5 minutes. Parents were in separate rooms and could not hear one another speak. They were asked to respond to the open-ended prompt: “*I*’*d like to hear your thoughts and feelings about (child*’*s name), in your own words and without my interrupting with any questions or comments. When I ask you to begin, I*’*d like you to speak for 5 minutes, telling me what kind of person (child*’*s name) is and how the two of you get along together. After you begin to speak, I prefer not to answer any questions until after the 5 minutes. Do you have any questions before we begin?*” Study personnel were not permitted to speak during the 5 minutes. The open-ended nature of the FMSS is meant to reduce response bias. All responses were recorded and a trained FMSS rater who was blind to the study questions coded the responses. The rater had high inter-rater reliability with 12 other FMSS trained raters (*M* = 93% [range = 80–100%]). Research has indicated that FMSS ratings of warmth and criticism are associated with observed behaviors within actual parent–child interactions (Weston et al., 2017). Additionally, the FMSS has been found to demonstrate high reliability and construct validity for families with autistic children (Greenberg et al., 2006).

For the present study, mothers spoke an average of 652.92 (*SD* = 151.73) words, and fathers spoke an average of 591.78 (*SD* = 181.75) words during the FMSS. Pearson correlations revealed that word count at T1 was not significantly associated with any of the study’s T1 main variables for mothers (CBCL: *r* = −.021, *p* = .798; CES-D: *r* = −.042, *p* = .608, positive remarks:*r* = .110, *p* = .185; critical comments: *r* = .039, *p* = .641) or fathers (CBCL: *r* = −.006, *p* = .940; CES-D: *r* = .068, *p* = .417; positive remarks: *r* = .111, *p* = .179; critical comments: *r* = .009, *p* = .916).

#### Warmth

For the current study, warmth was measured as the number of positive remarks made during the FMSS per recommendations from Magaña and colleagues (1986). A positive remark was defined as a statement that describes a positive characteristic of the child, a statement that compliments or praises the child, or a statement that expresses something desirable or enjoyable about the child. An example quote high in positive remarks from a mother-report FMSS is, “*[child*’*s name] is a great person. He*’*s so much fun. He*’*s so incredibly helpful* … *he*’*s very agreeable* … *he*’*s just really, really great* … *[he has] a funny sense of humor* … *I think we have a great relationship*”. An example quote from a father-report FMSS is, “*[child*’*s name] is my guy* … *. Um I really like [child*’*s name], he*’*s as sweet as they come. He*’*s a very good boy. He is very kind, very curious, um, very smart* … *and um yeah he*’*s pretty awesome, I really love [child*’*s name].*

#### Criticism

Criticism was coded as the total number of critical comments made during the five minutes (Magaña et al., 1986). Examples of comments coded as critical include: (1) describing the parent–child relationship negatively; and (2) indication of dissatisfaction within the parent–child relationship. Critical comments express disapproving, criticizing, or disappointing thoughts toward the child. An example quote involving criticism from a mother-report FMSS is, “*Um I think our relationship could be better* … *Um, when [child*’*s name] is in the picture, which is always, there*’*s a lot of tension between everybody. He is very needy, and needs this and needs that, and he can*’*t do anything on his own. He*’*s afraid of everything* … *Um and I felt angry with him, and frustrated and trapped that sometimes I don*’*t know what to do.*” An example quote from a father-report FMSS is, “*uh she irritates me at the same time. She*’*s disappointing in certain ways* … *um her inability to be regular or normal or intellectually anywhere is so disappointing* … *. I don*’*t know, her tactile abilities and inabilities are annoying.*”

### Data analysis plan

Descriptive statistics and boxplots were used to examine the variable distributions. Attrition analyses were conducted to explore whether families who completed all time points differed in parent depression symptoms, child mental health problems, or parent–child relationship quality from parents who had missing data. Bivariate Pearson correlations were conducted to assess the relatedness among the main study variables and with family sociodemographics. Family sociodemographics significantly correlated with main study variables were included as covariates in models. Specifically, we regressed the main study variables on the relevant family sociodemographic variables, and the unstandardized residual scores were entered into the model.

**Figure 1.**
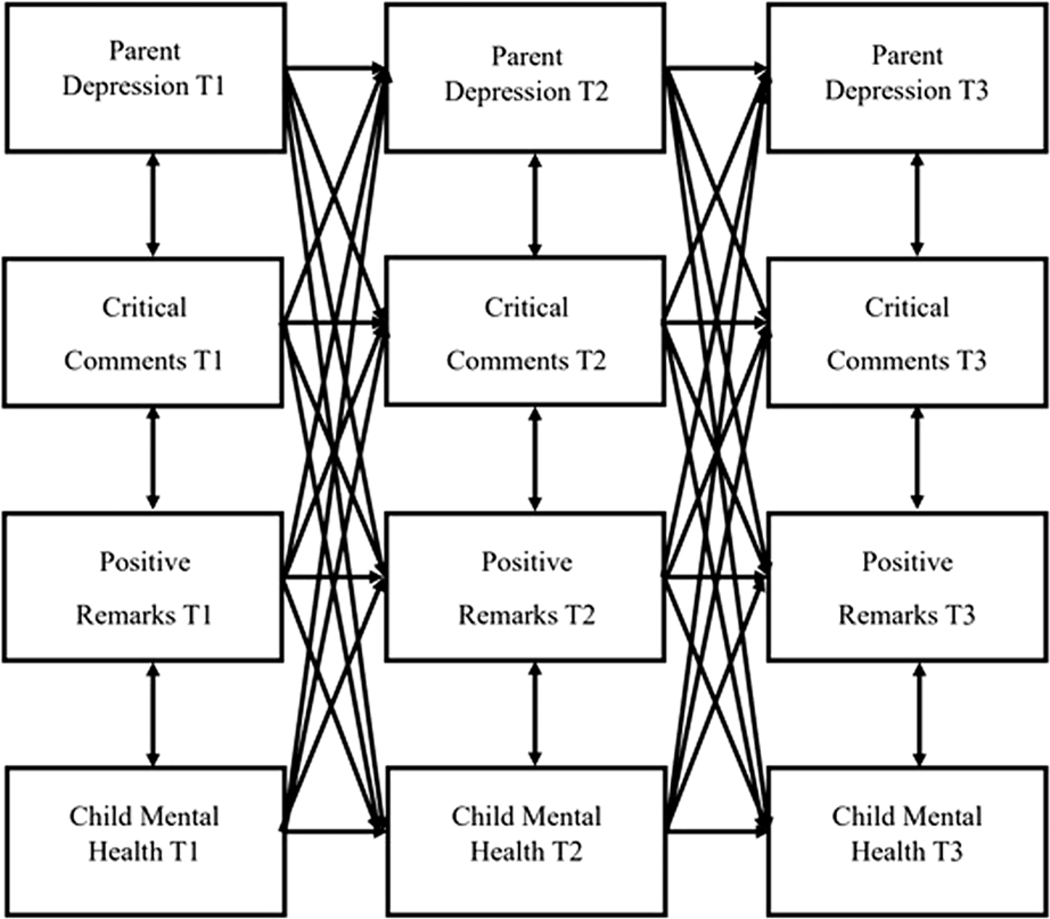
Simplified conceptual model of complete longitudinal mediation model.

For the primary model, we conducted an exploratory multigroup complete longitudinal mediation model using structural equation modeling (SEM). Figure 1 provides a simplified (direct effects only) conceptual model illustrating specified pathways of interest, and Supplemental Figure 1 depicts the full conceptual model (direct effects, lagged effects, cross-sectional associations). Initially, to examine if there was evidence for mother and father differences (at a global level), we analyzed a baseline model in which we tested equality constraints across mothers and fathers. More specifically, we tested a model in which all regression paths were constrained to be equal across groups compared to a model in which all paths were allowed to differ. Due to the results of the baseline model comparisons, potential differences in the dynamics of parent–child relationships for mothers versus fathers, and previous research indicating higher depression symptoms in mothers (versus fathers) of autistic children (Nunnally et al., 2023), the models were grouped by parent gender, providing separate results for mother- and father-reported measures and processes. Following the commonly implemented ratio of cases/observations to estimated parameters (10:1) and aligning with recommendations provided by Kline (2015) and Nunnally (1967), the minimum total sample size to detect a meaningful effect for each model is 240, and the minimum sample for each group (e.g., mothers and fathers) is 100. Within the complete longitudinal mediation model, we investigated: (1) whether criticism (in FMSS) mediated the association between parent depression symptoms and child mental health problems; and (2) whether warmth (in FMSS) mediated the association between parent depression symptoms and child mental health problems. Although our primary focus is on the mediation effect of parent–child relationship quality in our model, by using a complete longitudinal mediation model, as opposed to a focused mediation model, we were able to examine multiple potential mediation pathways occurring over time. This approach allowed us to test our specific hypotheses as defined above while also being able to explore other or additional potential longitudinal processes that might inform future research. All three variables of interest (e.g., parent depression symptoms, warmth/criticism, and child mental health problems) were represented in the model at T1–T3 and we tested primary and alternative mediational pathways for both parent and child-driven effects across the time points (Jose, 2016). Rather than constraining certain pathways across time, we estimated all possible pathways freely. This rationale was based on literature suggesting that the effects of parent depression and parent–couple relationship quality on child mental health (and vice versa) are not stagnant, but differ across child developmental stages (e.g., Goldberg & Carlson, 2014; Li et al., 2021; Myers et al., 2023).

The complete longitudinal mediation model was run in Mplus statistical software (Muthén & Muthén, 2012), as it allows for the examination of multiple mediation pathways (Jose, 2016). To analyze model fit, the root mean square error of approximation (RMSEA), comparative fit index (CFI), Tucker-Lewis Index (TLI), and chi-square (*χ*^2^) were examined. A RMSEA value between .05 and .08, CFI and TLI scores greater than .90 were used to indicate good model fit (Hu & Bentler, 1999; Little, 2013). For the purposes of our exploratory approach, we were interested in model fit indices to ensure our model was not excluding meaningful effects. The output provided one set of model fit indices for the multigroup model. The full information maximum likelihood method was implemented to account for missing data (Little, 2013), which is a better estimation method compared to deletion or imputation methods and is a robust estimator in SEM (Schlomer et al., 2010). We analyzed bias-corrected bootstrapped confidence intervals based on 5,000 iterations to determine if significant mediations are present within the model (Jose, 2013).

## Results

### Preliminary analyses

Parent depression symptoms and child mental health problems were approximately normally distributed with acceptable skew values (skew range for CES-D = 1.160 to 1.271; −.601 to −.414 for CBCL). At T1, data from both parents was available for 156 families. Six additional families provided mother-report only, for a total of 162 families (*N* = 318 parents). Of these families, at T2, data from both parents was available for 133 families. Four additional families provided mother-report only, for a total of 137 families (*N* = 270 parents). T3 included data from both parents for 122 families, with 3 additional families providing mother-report only, for a total of 125 families (*N* = 247 parents). Reasons for attrition included moving or not having time to participate at that time point. Mothers who completed all time points used more critical comments than mother “non-completers” (*t*(145) = 1.817, *p* = .036), and fathers who completed all time points used fewer positive comments than father “non-completers” (*t*(146) = −2.258, *p* = .013). Additionally, mother “completers” were younger than mother “non-completers” (*t*(162) = −1.899, *p* = 030), and father “completers” reported a higher income than “non-completers” (*t*(161) = 2.104, *p* = .018). Despite these findings, missing completely at random (MCAR) tests failed to reject MCAR (MCAR: *X*^2^ = 2.611, *p* > .05), suggesting that overall missingness was largely occurring completely at random.

Table 2 provides the means and standard deviations for main study variables as well as within-couple differences in mother-versus father reports. Paired sample *t*-tests revealed that mother- and father-reported CBCL scores did not differ significantly at any time point (*t*: 1.127–1.913, *p* > .05). Mother and father CES-D scores differed significantly at each time point (*t*s: 2.199–3.103, *p* < .05) with mothers reporting higher levels of depression symptoms than fathers. Mothers and fathers differed significantly on the number of critical comments at T1 (*t*(145) = 2.261, *p* = .025), with mothers reporting more critical comments than fathers, but no significant difference was found for T2–T3 (*t*s: 1.654–1.679, *p* > .05). Mothers and father’s differed significantly in their number of positive remarks for T1 and T3 (*t*s: 2.719–3.290, *p* < .05), with mothers reporting more positive remarks than fathers, but no differences were found at T2 (*t*(120) = .118, *p* = .906).

Table 3 shows Pearson correlations between parent depression symptoms, child mental health problems, critical comments, and positive remarks, as well as with family sociodemographic variables. Parent depression symptoms and child mental health problems were significantly positively correlated for both mother- (*rs* = .217–.460, *p* < .05) and father report (*rs* = .167–.434, *p* < .05) across all time points. Mother-report of critical comments was significantly positively associated with child mental health problems (*rs* = .270–.329, *p* < .05) and parent depression symptoms (*rs* = .209–.347, *p* < .05). Mother-report of positive remarks were significantly negatively associated with child mental health problems (*r* = −.243 – −2.04, *p* < .05) and parent depression symptoms (*rs* = −.250 – −.225, *p* < .05).

Father-reported critical comments were negatively associated with child mental health problems (*rs* = .204–.312, *p* < .05) and father-reported positive remarks was significantly negatively associated with child mental health problems at all time points (*r*s = −.355 – −.220, *p* < .05). Neither critical comments nor positive remarks were significantly correlated with parent depression symptoms for father reports.

Father age was positively correlated with child mental health problems (T1: *r* = .193, *p* = .016). Child ID status was significantly associated with father report of child mental health problems (*rs* = .188–.201, *p* < .05) and father-reported positive remarks (T1: *r* = −.242, *p* = .003). Household income was significantly correlated with parent depression symptoms for both mothers (T1: *r* = −.163, *p* = .038) and fathers (*rs* = −.283 – −.157, *p* < .05). The complete longitudinal mediation models controlled for parent age, child ID status, and income. Neither family size nor the presence of additional autistic children within a family were significantly associated with any of the main study variables (*p* > .05).

Prior to running the full mediation models, we ran a simplified model without the inclusion of a mediator. Child mental health problems predicted parent depression symptoms for both mothers (T1–T2: *β* = .278, *p* = .000; T2–T3: *β* =.194, *p* = .005) and fathers (T1–T2: *β* = .237, *p* = .002). Additionally, when examining potential differences between mothers and fathers, the model in which paths were allowed to differ was significantly different from the baseline model in which the paths were constrained (*p* < 0.01), indicating significant differences across groups, and supporting the need to examine mother and father coefficients separately.

### Complete-longitudinal mediation model

**Figure 2.**
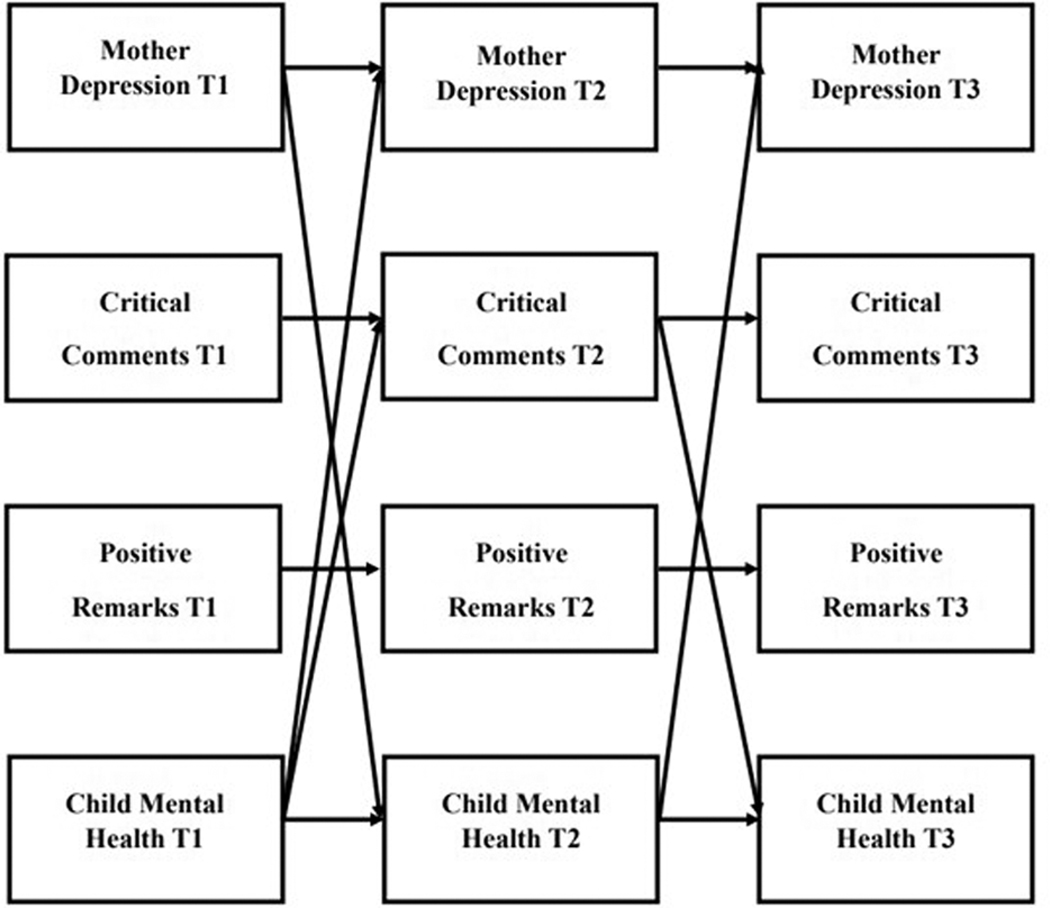
*Simplified* (showing direct effects only) results of the complete longitudinal mediation model for *mother-report* of mother depression symptoms, critical comments, positive remarks, and child mental health problems, controlling for parent age, household income, and child intellectual disability status. (Nonsignificant paths removed). Values are standardized path estimates. β-values and standard errors for direct effects are provided in Table 4. Lagged paths and cross-sectional associations removed for simplicity.

**Figure 3.**
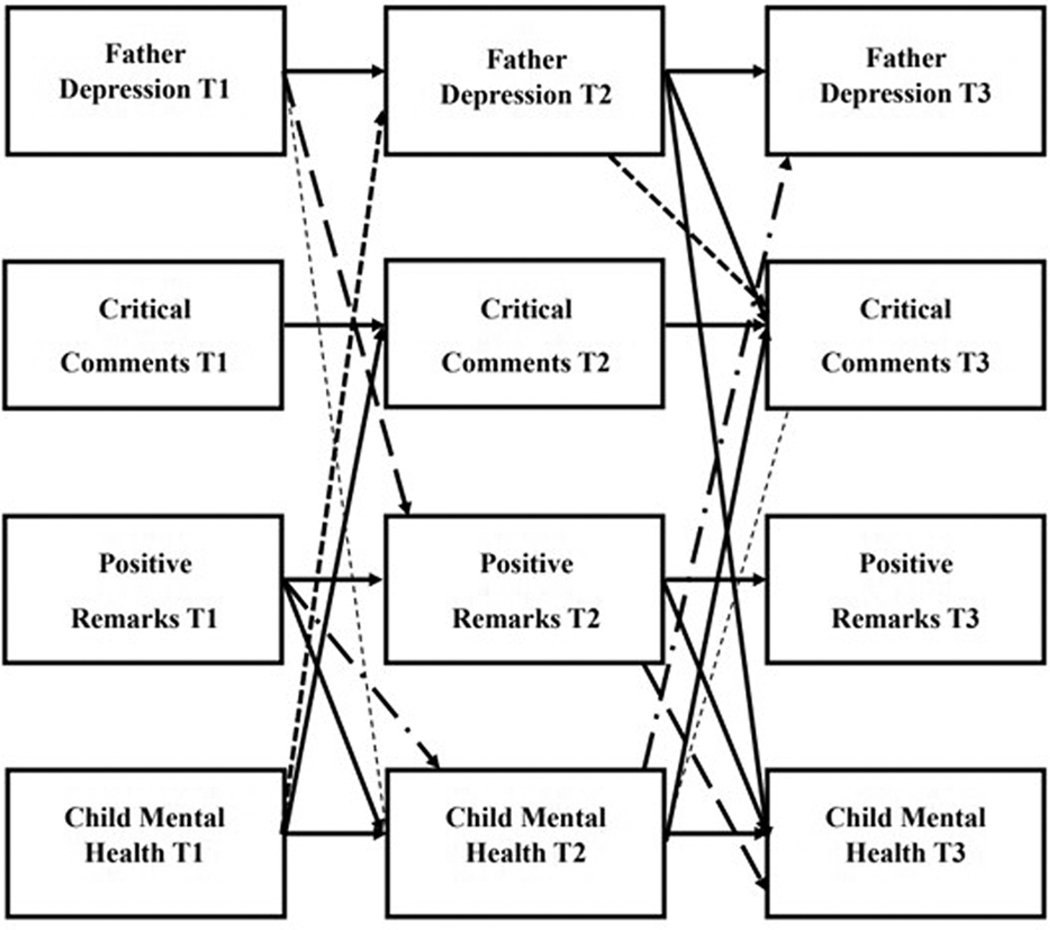
*Simplified* results (showing direct and indirect effects) of the complete longitudinal mediation model for *father-report* of father depression symptoms, critical comments, positive remarks, and child mental health problems, controlling for parent age, household income, and child intellectual disability status. (Nonsignificant paths removed). Values are standardized path estimates. The various dashed lines indicate a significant indirect pathway. β-values and standard errors for direct and indirect effects are provided in Tables 4 and 5, respectively. Lagged paths and cross-sectional associations removed for simplicity.

Figures 2 and 3 depict significant direct and indirect effects for mother- and father reports of complete-longitudinal mediation model, respectively. Supplemental Figures 2 and 3 illustrate mother- and father reports of the full model including all significant lagged and cross-sectional effects, respectively. Tables 4 and 5 provide the path coefficients for the direct and indirect pathways for these models. The model revealed good model fit (*X*^*2*^ (32) = 33.647, *p* = 0.3876; RMSEA = .018; TLI = 0.994; CFI = 0.998). Stability effects across time were present for mother- and father report of depression symptoms (Mother: T1–T2 *β* = 0.522, *p* = 0.000; T2–T3 *β* = .717, *p* = .000; T1–T3 *β* = 0.450, *p* = .000; Father: T1–T2 *β* = 0.455, *p* = 0.001; T2–T3 *β* = 0.606, *p* = 0.000), child mental health problems (Mother: T1–T2 *β* = 0.697, *p* = 0.000; T2-T3 *β* = 0.788, *p* = 0.003; Father: T1–T2 *β* = 0.752, *p* = 0.000; T2–T3 *β* = 0.833, *p* = 0.00; T1–T3 *β* = 0.252, *p* = 0.014), critical comments (Mother: T1–T2 *β* = 0.386, *p* = 0.004; T3–T4 *β* = 0.308, *p* = 0.001; T1–T3 *β* = 0.198, *p* = 0.002; Father: T1–T2 *β* = 0.407, *p* = 0.000; T2–T3 *β* = 0.302, *p* = 0.004; T1–T3 *β* = 0.137, *p* = 0.004), and positive remarks (Mother: T1–T2 *β* = 0.547, *p* = 0.000; T2–T3 *β* = 0.346, *p* = 0.013; Father: T1–T2 *β* = 0.468, *p* = 0.000; T2–T3 *β* = 0.305, *p* = 0.000; T1–T3 *β* = 0.400, *p* = 0.002). There were also significant cross-sectional associations between parent depression symptoms and child mental health problems, positive remarks, and critical comments across the same time points in expected directions. Beta coefficients and *p* values for cross-sectional pathways can be found in Supplemental Table 1.

### Direct effects

Mother-report of child mental health problems directly predicted increased maternal depression symptoms 12 months later (T1 CBCL to T2 CES-D: *β* = .252, *p* = .000; T2 CBCL to T3 CES-D: *β* = .269, *p* = .000), and in the opposite direction, mother depression symptoms at T1 predicted greater child mental health problems at T2 (*β* = 0.053, *p* = 0.001). Additionally, mother-report of child mental health problems at T1 directly predicted maternal critical comments at T2 (*β* = .240 *p* = .021). In the opposite direction, mother critical comments at T2 directly predicted child mental health problems at T3 (*β* = −.078, *p* = .004).

Father report of child mental health problems directly predicted an increase in the number of critical comments used to describe the parent–child relationship (T1–T2: *β* = 0.183 *p* = 0.045; T2–T3: *β* = 0.164 *p* = 0.003). Further, father depression symptoms at T2 predicted number of critical comments at T3 (*β* = −0.078, *p* = 0.005) as well as child mental health problems (T2–T3: *β* = 0.167, *p* = 0.000) 12 months later. Additionally, father report of positive remarks predicted a decrease in child mental health problems 12 months later (T1–T2: *β* = −0.075, *p* = 0.008; T2–T3: *β* = −0.171, *p* = 0.004).

### Indirect effects

Indirectly, father report of child mental health problems at T1 predicted paternal depression symptoms at T2, which then predicted the number of father critical comments at T3 (*β* = −0.017, *p* = 0.018; CI [−0.023 – −0.015]). This finding suggests that parent depression symptoms partially mediated the connection between child mental health problems and father number of critical comments. Additionally, father depression symptoms at T1 predicted greater child mental health problems at T2, which then predicted a greater number of critical comments at T3 (*β* = 0.016, *p* = 0.040; CI [0.003–0.023]), which suggests that child mental health problems partially mediated the association between father depression symptoms and the number of critical comments about the child. Additionally, father depression symptoms at T1 predicted father’s number of positive remarks at T2, which then predicted child mental health problems at T3 (*β* = 0.022, *p* = 0.006; CI [ 0.019–0.034]). This finding suggests that positive remarks mediated the association between higher father depression symptoms and higher child mental health problems. Finally, father report of positive remarks at T1 predicted child mental health problems at T2, which then predicted father depression symptoms at T3 (*β* = −0.009, *p* = 0.032; CI [−0.010 – −0.009]), suggesting that child mental health problems served as a mediator between number of positive remarks and father depression symptoms.

#### Secondary analyses

Our model revealed two unexpected findings that were explored in secondary analyses. There was an unexpected direct negative effect of T2 mother critical comments on T3 child mental health problems (*β* = −.078, *p* = .004). However, further investigation highlighted a suppression effect (Maassen & Bakker, 2001), which can occur in longitudinal models when simultaneously evaluating multiple predictors rather than each pathway in isolation, often reflecting measurement issues. Specifically, the high correlation among child mental health problems at all time points appeared responsible for a suppression effect (i.e., β value switches direction when adding predictor(s)) (Maassen & Bakker, 2001). When multiple linear regressions were conducted to examine the effects of T1-T2 CBCL, T2 critical comments and T2 CES-D on T3 CBCL, the β of mother critical comments predicting T3 child mental health problems was weak and nonsignificant (*β* = −0.020, *p* = .683). After removing T1 and T2 CBCL scores, the β value switched back to positive and became significant (*β* = .300, *p* = .001), indicating that T2 critical comments predicted *higher* T3 child mental health problems.

A second suppression effect was found for the unexpected association between father report of parent depression symptoms at T2 and fewer T3 critical comments. Multiple linear regression models examining the effects of T1-T2 critical comments, T2 CES-D and T2 CBCL on T3 critical comments revealed that the β value for T2 CES-D became positive and significant (*β* = .244, *p* < .001), suggesting that parent depression symptoms at T2 predicts an *increase* in the number of critical comments. Due to this suppression effect being part of the significant father-reported *indirect* pathway (i.e., T1 child mental health problems **➔** T2 parent depression symptoms **➔** T3 critical comments), conducted the Sobel Test (Sobel, 1982) in SPSS. These results suggested that T2 parent depression symptoms partially mediated the association between T1 child mental health problems and T3 critical comments (Path A: *β* = .422, *SE* = .070; Path B: *β* = .010, *SE* = .004; Path C: *β* = .014, *SE* = .005; Sobel Test: *z* = 2.31, *SE* = .002, *p* = .021).

## Discussion

Families are interactive and reactive (Cridland et al., 2014), such that the emotions and behaviors of one member or subsystem influence those of other members and subsystems within the family. The goal of the present study was to understand the role of the parent–child relationship in driving longitudinal and bidirectional associations between parent depression and the mental health problems of autistic children. However, given the intertwined nature of the family system, and the lack of prior longitudinal data on these mediation pathways in an autism sample, complete longitudinal mediation models were conducted to allow us to capture alternative mediation pathways that could be occurring.

In our study of 162 families of autistic children, we found evidence for robust connections between parent depression symptoms, child mental health problems, and parent–child relationship quality (for both criticism and warmth). These pathways were in line with our primary hypotheses. Specifically, mother-report of child mental health problems predicted an increase in mother depression symptoms 12 months later (from T1→T2 and T2→ T3). Both mother- and father report of T1 child mental health problems predicted an increase in their critical comments about their autistic child and parent–child relationship at T2. These pathways align with research outside of autism (Chan et al., 2018; Crugnola et al., 2016; Mackler et al., 2015; Stone et al., 2016) and suggest that child mental health problems can alter parent mood and may contribute to critical parent–child interactions. In the opposite direction, both mother and father depression symptoms predicted an increase in the mental health problems of the autistic child across time (Mothers: T1→T2; Fathers: T2→T3). This finding is consistent with previous research that exposure to parent depression negatively affects child mental health (Cohen & Tsiouris, 2006; Wiggins et al., 2019). It is also possible that depressed parents have a biased perception of their child’s mental health, as parent perception can be influenced by their own mental health (Abate et al., 2018, Kroes et al., 2003; Ringoot et al., 2015). Interestingly, in the present study, mothers reported significantly higher levels of depression symptoms than fathers; however, our models indicate that paternal depression symptoms had a greater effect on both child mental health and the parent–child relationship (for both critical comments and positive remarks) than maternal depression symptoms. Thus, despite having less severe depressive symptoms on average than mothers, father depression symptoms appear to take a greater toll on the parent–child relationship and child mental health than mothers’ depressive symptoms.

We also found evidence of significant mediation pathways. Specifically, higher T1 child mental health problems were related to higher T3 father critical comments, and this was partially mediated by higher T2 father depression symptoms. Moreover, there was a positive association between T1 father depression symptoms and T3 father critical comments that was partially mediated by higher T2 child mental health problems. These pathways suggest that the mental health of fathers and their autistic child are reciprocally associated in a feedback loop that contributes to a more critical parent–child relationship across time. As hypothesized, positivity in the parent–child relationship also mediated connections between parent and child mental health. Specifically, for fathers, there was a positive association between T1 parent depression symptoms and T3 child mental health problems that was mediated by decreased positive remarks about the child and parent–child relationship at T2. This pathway suggests that fathers with high depression symptoms had a less positive parent–child relationship over time, which has subsequently added to the autistic child’s mental health problems. Additionally, father positive remarks at T1 were significantly associated with T3 father depression symptoms, which was partially mediated by child mental health problems at T2. Evidence for similar pathways of effects has been found outside of autism (Aktar & Bögels, 2017; Rueger et al., 2011), and points to the parent-child relationship as a potential target for the prevention of both child and parent mental health symptoms. Helping parents, and specifically fathers, increase positivity (as opposed to decreasing negativity) within their relationship may have a positive downstream effect on child mental health, and in turn, lead to reductions in father depression symptoms. Previous research on the father–child relationship in the context of autism highlights the important role fathers play in child development and suggest it may be useful to tailor interventions directed at fathers specific to their unique interactional style (see Perzolli et al., 2021 for a review).

It is not clear why mediation pathways tended to be statistically significant for father-reported models but not mother-reported models of the parent–child relationship. Autistic children may be more sensitive to both criticism and positivity in the father–child relationship than in the mother-child relationship. Evidence is mixed as to whether mothers and fathers interact differently with their autistic children, with some evidence showing clear differences in communication and play style between mothers and fathers of autistic children and others showing larger differences in comparison to parents of neurotypical children (Perzolli et al., 2021). Perhaps, this is because fathers, on average, take on fewer childcare responsibilities than mothers within families of autistic children (Hartley et al., 2014; Sharabi & Marom-Golan, 2018). Critical or warm interactions, even if infrequent, may take a greater toll on both children and parents without a context of frequent neutral interactions. Additional research is needed to clarify whether and how father–child interactions may differ compared to mother-child dyads and how such differences (and similarities) may be useful during intervention.

### Strengths, limitations, and future directions

The present study had several strengths including the use of complete longitudinal mediation modeling using structural equation modeling which allowed us to simultaneously explore multiple parent- and child-driven pathways of effects. This analytic approach provided a complete picture of numerous time-ordered associations between parent depression symptoms, parent–child relationship quality, and child mental health problems, mirroring theoretical models of complex and bidirectional family dynamics (e.g., Jose, 2016). Another study strength was the inclusion of both mother and father experiences through a group variable in the structural equation models. There is a paucity of father perspectives in family research, and more specifically, in autism research and thus the current findings build on this literature and also highlight important differences in the experiences of mothers and fathers.

The current study also had limitations. The generalizability of findings is limited to the sociodemographics of the families included. Our sample largely consisted of White, non-Hispanic parents and those of middle socioeconomical class and heterosexual couples. Additionally, in order to participate in the current study, parent-couples needed to be with their current partner for a minimum of three years and currently be cohabiting. It is possible that this inclusion criteria created a selection effect in that parent-couples who separated/divorced (perhaps due to severe parent depression or related to having a child with more severe autism symptoms) prior to their autistic child being aged 6–13 years (and did not recouple) are not represented in our study. As a result, our findings may represent families who are less severely impacted by depression, experienced low child related stressors, and/or who had longstanding couple relationships. In addition, families who completed all three waves of the study and included in analyses were younger and had higher income, on average, than those who originally enrolled in the study but did not complete all three time points. While we did not find differences in reports of parent depression symptoms or child mental health problems between “completers” and “non-completers,” the families who completed all three study waves had lower quality parent–child relationships (fewer maternal positive remarks and more paternal critical comments) than those who did not. It is possible that families who were satisfied with their parent–child relationship quality were not as motivated to engage in research to improve services and supports. Due to the lack of father perspectives in family research, the present study focused on mother and father perspectives; however, future research should include a diverse array of families (e.g., same-sex couples and single parents).

In the current study, it is also possible that depressed parents rated their parent–child relationship or child mental health symptoms more negatively due to their own depression. Thus, future studies should include observations of actual parent–child interactions and ratings of child mental health problems from alternative informants (e.g., self-report, teacher-report, etc.). Of note, effect sizes in the current study were small. Future replication of the complete longitudinal mediation model should be conducted in larger nonrelated samples. Finally, twenty families from the present study had at least one additional autistic child which may have also added to parent depression or the target child’s mental health problems.

### Study implications

Left untreated, mental health problems in parents or autistic children influence one another by taking a toll on the parent–child relationship. There is a need for research to identify efficacious family-wide interventions aimed at improving the mental health of parents and autistic children and supporting positive parent–child relationships. Strategies that intervene on parent and/or child mental health such as mindfulness training or cognitive behavioral therapy may also be beneficial in improving the parent–child relationship. Future research should identify how adaptive family processes can be leveraged to improve mental health in families of autistic children.

## Supplementary Material

1

2

3

4

## Figures and Tables

**Table 1.** Family sociodemographics

Demographic	*M* (*SD*)
Mother (*n* = 162)	
Age in years (*M* [*SD*])	39.76 (5.61)
Race/Ethnicity (N [%])	
White, non-Hispanic	148 (91)
Other	14 (9)
Father (*n* = 156)	
Age in years (*M* [*SD*])	40.76 (6.19)
Race/Ethnicity (N [%])	
White, non-Hispanic	138 (89)
Other	18 (11)
Parent Couple	
Household Income (N [%])	
$1-$9,999	2 (1)
$10,000–$19,999	3 (2)
$20,000–$29,999	7 (4)
$30,000–$39,999	7 (4)
$40,000–$49,999	7 (4)
$50,000–$59,999	10 (6)
$60,000–$69,999	19 (12)
$70,000–$79,999	20 (12)
$80,000–$89,999	17 (10)
$90,000–$99,999	13 (8)
$100,000–$119,999	19 (12)
$120,000–$139,999	15 (9)
$140,000–$159,999	11 (7)
$160,000 +	12 (7)
Couple Relationship Length, years (*M* [*SD*])	14.55 (5.58)
Number of Children (M [SD])	2.43 (1.04)
Families with Additional Autistic Child (N [%])	20 (12)
Target Child (*n* = 162)	
Male (N [%])	140 (86)
Age in years (*M* [*SD*])	9.03 (2.25)
ID (N [%])	57 (35)

*Note. M* = mean; *SD* = standard deviation; *N* = sample size; ID = intellectual disability.

**Table 2.** Mother & father reported means, standard deviations, and t-values for main variables

Measure	Mother *M*^1^(*SD*)^2^	Father *M*(*SD*)	*t*-value^3^	*df* ^4^	*p*-value
Time 1	*n* = 162	*n* = 156			
CES-D^5^ Total	18.40 (7.10)	16.44 (6.40)	3.029	154	.003**
CBCL^6^ Total T	63.83 (8.71)	62.80 (9.33)	1.701	156	.091
Critical^7^	0.35 (0.88)	0.18 (0.52)	2.261	145	.025*
Positive^8^	3.55 (2.96)	2.72 (2.60)	3.290	145	.001**
Time 2	*n* = 137	*n* = 133			
CES-D Total	14.86 (11.29)	11.14 (9.51)	3.103	130	.002**
CBCL Total T	63.14 (9.01)	61.95 (8.92)	1.913	131	.058
Critical	0.35 (0.66)	0.23 (0.69)	1.679	120	.096
Positive	3.07 (3.05)	3.02 (3.12)	.118	120	.906
Time 3	*n* = 125	*n* = 122			
CES-D Total	14.68 (10.70)	12.40 (10.41)	2.199	116	.030*
CBCL Total T	62.79 (8.70)	62.06 (9.64)	1.127	120	.262
Critical	0.31 (0.69)	0.20 (0.47)	1.654	102	.101
Positive	3.24 (2.88)	2.44 (2.33)	2.719	102	.008**

Note.

1 mean

2 standard deviation

3 value for paired-samples *t*-test

4 degrees of freedom

5 Center for Epidemiological Studies-Depression Scale total score (Radloff, 1977)

6 Child Behavior Checklist Total Problems T-Score (Achenbach & Rescorla, 2001)

7 number of critical comments from the Five Minute Speech Sample (Magaña et al., 1986)

8 number of positive remarks from the Five Minute Speech Sample (Magaña et al., 1986)

* *p* < .05

** *p* < .01.

**Table 3.** Correlations between main study variables and sociodemographics

	1	2	3	4	5	6	7	8	9	10	11	12	13	14	15	16	17
1. P.Age^1^	−	−.025	.097	−.119	−.004	−.065	.010	−.157	.153	.087	.084	−.040	−.006	.186^†^	−.020	−.025	−.022
2. Inc.^2^	−.011	−	−.183*	−.015	−.013	−.163*	−.045	−.051	.039	−.019	.052	−.094	−.148	−.097	.114	.033	−.050
3. ID^3^	.033	−.183*	−	.058	.000	.043	−.053	.063	.113	.161	.151	−.073	−.022	−.065	−.012	−.124	.009
4. FSize^4^	−.046	−.015	.058	−	.169*	−.023	−.033	−.014	−.065	−.029	−.116	−.056	−.132	−.047	.086	.076	−.017
5. sASD^5^	.067	−.015	.002	.169*	−	.020	.094	.081	.083	.091	.091	.039	−.058	.042	−.026	−.021	−.091
6. Dep^6^1	.063	−.157*	−.024	−.002	−.025	−	.589**	.691**	.267**	.246**	.217**	−.002	−.030	−.016	.209*	.086	.172
7. Dep2	.073	−.124	−.010	−.120	−.046	.524**	−	.717**	.413**	.460**	.366**	−.160	−.250**	−.225**	.347**	.290**	.318**
8. Dep3	.037	−.283**	−.130	−.041	.046	.429**	.736**	−	.411**	.406**	.389**	−.046	−.153	−.174	.325**	.225*	.285**
9. CB1^7^	.193*	−.112	.188*	−.094	.104	.167*	.273**	.247**	−	.783**	.792**	−.228**	−.165	−.126	.312**	.329**	.270**
10. CB2	.158	−.104	.048	−.172	.054	.247**	.434**	.342**	.777**	−	.830**	−.232**	−.204*	−.142	.295**	.282**	.327**
11. CB3	.100	−.071	.201*	−.016	.177	.193*	.354**	.315**	.809**	.833**	−	−.243**	−.232*	−.223*	.280**	.300**	.307**
12. Pos^8^1	−.072	.083	−.242**	−.056	.007	−.043	−.083	−.029	−.348**	−.310**	−.343**	−	.549**	.231*	−.298**	−.246**	−.191*
13. Pos2	−.173	.050	−.015	−.055	−.043	−.113	−.106	−.039	−.220*	−.355**	−.326**	.434**	−	.400**	−.211*	−.296**	−.181
14. Pos3	.003	.048	.001	−.093	−.030	−.085	−.101	−.180	−.328**	−.315**	−.334**	.479**	.458**	−	−.153	−.165	−.246**
15. Crit^9^1	.025	.017	−.004	−.059	.042	.099	.070	.081	.138	.149	.240**	−.155	−.190*	−.067	−	.469**	.458**
16. Crit2	.008	−.103	−.048	−.100	−.044	.111	.013	.056	.178	.207*	.225*	−.271**	−.224*	−.212*	.604**	−	.308**
17. Crit3	−.001	.015	−.122	−.002	.058	−.043	.043	.134	.248*	.204*	.312**	−.147	.246*	−.301**	.322**	.460**	−

*Note.* Pearson Correlations. Mother−report is shaded and above the diagonal. Father−report of unshaded and below the diagonal.

1 Parent age

2 Household Income

3 Intellectual Disability

4 Family Size (total number of children)

5 Sibling with ASD

6 Center for Epidemiological Studies−Depression Scale (Radloff, 1977)

7 Child Behavior Checklist (Achenbach & Rescorla, 2001);

8 Positive Remarks from the Five−Minute Speech Sample (Magaña et al., 1986)

9 Critical Comments from the Five−Minute Speech Sample (Magaña et al., 1986).

† = *p* < .10

* = *p* < .05

** = *p* < .01.

**Table 4.** Standardized and unstandardized path coefficients for mother-and father-reports of parent depression, critical comments, positive remarks, and child mental health problems

Time Point	Mother-report *B*^1^(*SE*^2^), Unstandardized	Mother-report *β*(*SE*), Standardized	Father-report *β*(*SE*), Unstandardized	Father-report *β*(*SE*), Standardized
**Cross Effects**	**CES − D**^3^ ➔ **Critical**^4^			
**1➔2**	−0.012(0.008)	−0.127(0.083)	0.000(0.005)	−0.001(0.064)
**2➔3**	−0.005(0.012)	−0.053(0.183)	−0.004(0.002)*	−0.078(0.028)*
	**CES − D ➔ Positive** ^5^			
**1➔2**	0.014(0.060)	0.030(0.131)	−0.067(0.039)	−0.129(0.078)
**2➔3**	−0.010(0.038)	−0.037(0.134)	0.002(0.023)	0.006(0.079)
	**CES − D ➔ CBCL** ^6^			
**1➔2**	0.074(0.033)*	0.053(0.016)*	0.143(0.101)	0.098(0.084)
**2➔3**	−0.106(0.054)*	−0.089(0.049)	0.260(0.070)**	0.167(0.034)**
	**Critical ➔ CES − D**			
**1➔2**	1.419(0.958)	0.109(0.081)	−0.217(2.657)	−0.012(0.148)
**2➔3**	−0.324(0.262)	−0.020(0.022)	1.196(3.616)	0.072(0.124)
	**Critical ➔ CBCL**			
**1➔2**	−0.173(0.396)	−0.016(0.038)	−0.504(1.190)	−0.030(0.071)
**2➔3**	−1.606(0.542)*	−0.078(0.027)*	−0.093(.863)	−0.004(0.021)
	**Positive ➔ CES − D**			
**1➔2**	0.011(0.206)	0.003(0.055)	−0.123(0.138)	−0.034(0.041)
**2➔3**	−0.126(0.437)	−0.036(0.121)	0.357(0.174)*	0.115(0.063)
	**Positive ➔ CBCL**			
**1➔2**	−0.091(0.139)	−0.027(0.047)	−0.255(0.095)*	−0.075(0.028)*
**2➔3**	0.110(0.240)	0.025(0.078)	−0.804(0.237)*	−0.171(0.059)*
	**CBCL➔ Critical**			
**1➔2**	0.019(0.010)	0.240(0.104)*	0.012(0.005)*	0.183(0.091)*
**2➔3**	0.050(0.028)	0.488(0.268)	0.009(0.003)*	0.164(0.054)*
	**CBCL ➔ Positive**			
**1➔2**	−0.004(0.040)	−0.011(0.109)	−0.003(0.050)	−0.008(0.136)
**2➔3**	−0.082(0.070)	−0.250(0.189)	−0.030(0.040)	−0.108(0.141)
	**CBCL ➔ CES − D**			
**1➔2**	0.339(0.035)**	0.252(0.045)**	0.231(0.131)	0.218(0.129)
**2➔3**	0.295(0.075)**	0.269(0.042)**	0.126(0.082)	0.115(0.060)
**Lagged Effects**	**CESD**			
**1➔3**	0.696(0.160)**	0.450(0.094)**	0.137(0.198)	0.085(0.109)
	**Critical**			
	0.225(0.042)**	0.198(0.065)*	0.128(0.045)*	0.137(0.048)*
	**Positive**			
	−0.084(0.124)	−0.077(0.100)	0.385(0.152)*	0.400(0.128)*
	**CBCL**			
	0.194(0.247)	0.121(0.206)	0.418(0.168)*	0.252(0.103)*

Note.

1 Beta value

2 Standard Error

3 Center for Epidemiological Studies−Depression Scale (Radloff, 1977)

4 Critical comments from the Five Minute Speech Sample (Magaña et al., 1986)

5 Positive remarks from the Five Minute Speech Sample (Magaña et al., 1986)

6 Child Behavior Checklist (Achenbach & Rescorla, 2001)

* *p* < .05

** *p* < .01.

**Table 5.** Estimates of indirect pathways for longitudinal mediation model

Time Point	Pathway	Mother-Report β^1-^value	SE^2^	Lower Bound of 95% CI^3^	Upper Bound of 95% CI	Father-Report *B*-value	SE	Lower Bound of 95% CI	Upper Bound of 95% CI
1**➔**2**➔**3									
	CES − D^4^**➔**Critical^5^**➔**CBCL^6^	.010	.007	−.001	.015	.000	.004	−.007	.002
	CES − D**➔**Positive^7^**➔**CBCL	.001	.006	−.009	.006	**.022** *	.008	.019	.034
	CES − D**➔**CBCL**➔**Critical	.026	.022	−.003	.036	**.016** *	.008	.003	.023
	CES − D**➔**CBCL**➔**Positive	−.013	.015	−.029	.005	−.011	.021	−.045	−.004
	Critical**➔**CBCL**➔**CES − D	−.004	.009	−.023	−.001	−.003	.009	−.016	.007
	Critical**➔**CES − D**➔**CBCL	−.010	.007	−.013	−.006	−.002	.023	−.032	−.002
	Positive**➔**CBCL**➔**CES − D	−.007	.014	−.024	.011	**−.009** *	.004	−.010	−.009
	Positive**➔**CES − D**➔**CBCL	.000	.005	.000	.007	−.006	.007	−.006	.003
	CBCL**➔**Critical**➔**CES − D	−.005	.010	−.025	−.008	.013	.018	−.014	.030
	CBCL**➔**Positive**➔**CES − D	.000	.017	−.023	.002	−.001	.016	−.025	.005
	CBCL**➔**CES − D**➔**Critical	−.013	.047	−.034	.068	**−.017** *	.007	−.023	−.015
	CBCL**➔**CES − D**➔**Positive	−.009	.037	−.047	.051	.001	.018	−.011	.032

*Note.* Estimates are standardized values. Significant values are bold.

1 Beta−value

2 Standard Error

3 Confidence Interval

4 Center for Epidemiological Studies−Depression Scale (Radloff, 1977)

5 Critical comments from the Five−Minute Speech Sample (Magaña et al., 1986)

6 Child Behavior Checklist (Achenbach & Rescorla, 2001)

7 Positive remarks from the Five−Minute Speech Sample (Magaña et al., 1986)

* *p* < .05.
