# Supplementary figures and images for: Longitudinal pathways between parent depression and child mental health in families of autistic children
Source: Dev Psychopathol. Author manuscript; Available in PMC 2025 Sep 5. (PMC11929618; doi:10.1017/S0954579424001378)

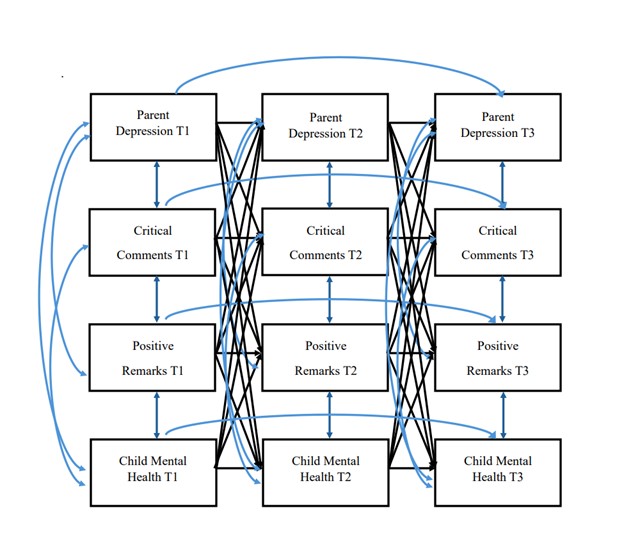

Supplement: 1 [file NIHMS2018453-supplement-1.zip › Supplemental_Figure_1.jpg]

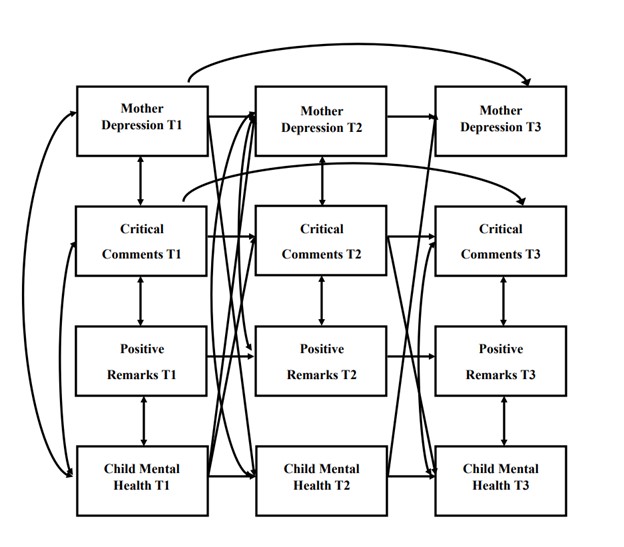

Supplement: 2 [file NIHMS2018453-supplement-2.zip › Supplemental_Figure_2.jpg]

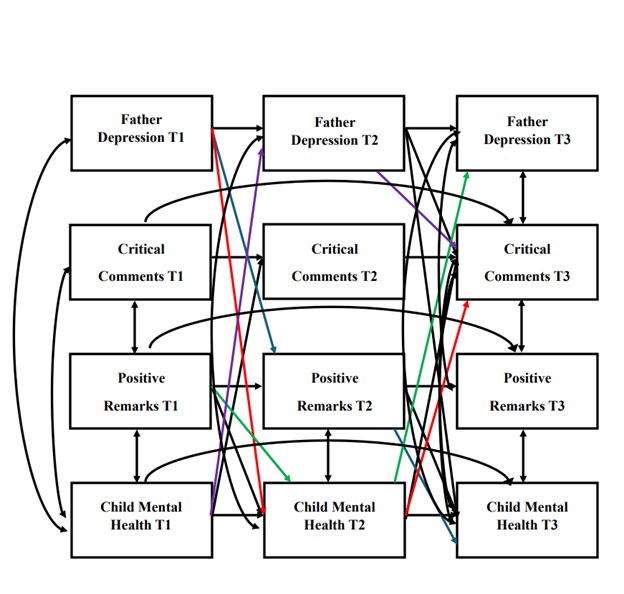

Supplement: 3 [file NIHMS2018453-supplement-3.zip › Supplemental_Figure_3.jpg]
