# Supplementary material for: Longitudinal pathways between parent depression and child mental health in families of autistic children
Source: Dev Psychopathol. Author manuscript; Available in PMC 2025 Sep 5. (PMC11929618; doi:10.1017/S0954579424001378)
Supplement: 4 [file NIHMS2018453-supplement-4.docx]

|  | Mothers | |  | Fathers | |  |
| --- | --- | --- | --- | --- | --- | --- |
|  | β | *p* | β | | *p* | |
| T1 |  |  |  | |  | |
| CES-D^1^ Positive^2^ | -0.008 | 0.943 | -0.077 | | 0.520 | |
| CES-D Critical^3^ | **0.235** | 0.016 | 0.113 | | 0.309 | |
| CES-D CBCL^4^ | **0.288** | 0.001 | **0.154** | | 0.003 | |
| Positive Critical | **-0.299** | 0.000 | **-0.179** | | 0.002 | |
| Positive CBCL | **-0.218** | 0.000 | **-0.316** | | 0.006 | |
| Critical CBCL | **0.329** | 0.000 | **0.163** | | 0.000 | |
| T2 |  |  |  | |  | |
| CES-D Positive | **-0.274** | 0.027 | -0.023 | | 0.744 | |
| CES-D Critical | **0.183** | 0.029 | 0.013 | | 0.883 | |
| CES-D CBCL | **0.194** | 0.003 | **0.346** | | 0.000 | |
| Positive Critical | **-0.204** | 0.000 | -0.034 | | 0.583 | |
| Positive CBCL | -0.121 | 0.093 | **-0.254** | | 0.019 | |
| Critical CBCL | 0.025 | 0.835 | 0.021 | | 0.659 | |
| T3 |  |  |  | |  | |
| CES-D Positive | -0.174 | 0.242 | **-0.246** | | 0.000 | |
| CES-D Critical | 0.177 | 0.271 | **0.183** | | 0.008 | |
| CES-D CBCL | 0.146 | 0.307 | **0.315** | | 0.000 | |
| Positive Critical | **-0.439** | 0002 | **-0.262** | | 0.000 | |
| Positive CBCL | **-0.524** | 0.000 | **-0.334** | | 0.000 | |
| Critical CBCL | **0.642** | 0.015 | **0.312** | | 0.001 | |

**Supplementary Table 1.** *Cross-Sectional Associations*

*Note.* ^1^Center for Epidemiological Studies-Depression Scale total score (Radloff, 1977); ^2^number of positive remarks from the Five-Minute Speech Sample (Magaña et al., 1986); ^3^number of critical comments from the Five-Minute Speech Sample (Magaña et al., 1986); ^4^Child Behavior Checklist Total Problems T-Score (Achenbach & Rescorla, 2001); Coefficients are standardized estimates. Significant values are in bold.
